# Pantoprazole blocks the JAK2/STAT3 pathway to alleviate skeletal muscle wasting in cancer cachexia by inhibiting inflammatory response

**DOI:** 10.18632/oncotarget.17387

**Published:** 2017-04-24

**Authors:** Dunwei Guo, Chaoyi Wang, Qiang Wang, Zhongpeng Qiao, Hua Tang

**Affiliations:** ^1^ Department of Gastrointestinal Surgery, The First Affiliated Hospital of Chongqing Medical University Chongqing 400016, China; ^2^ Department of Breast Surgery, Chongqing Three Gorges Central Hospital, Chongqing 404000, China; ^3^ Department of Gastrointestinal Surgery, Suining Municipal Central Hospital, Suining, Sichuan 629000, China

**Keywords:** cancer cachexia, JAK2/STAT3, ubiquitin, Fbx32, pantoprazole

## Abstract

**Objective:**

Cancer cachexia is often present in patients with advanced malignant tumors, and the subsequent body weight reduction results in poor quality of life. However, there has been no progress in developing effective clinical therapeutic strategies for skeletal muscle wasting in cancer cachexia. Herein, we explored the functions of pantoprazole on cancer cachexia skeletal muscle wasting.

**Methods:**

The mouse colon adenocarcinoma cell line C26 was inoculated in the right forelimb of male BALB/C mice to establish a cancer cachexia model. The animals were treated with or without different concentrations of pantoprazole orally, and the body weight, tumor growth, spontaneous activity, and muscle functions were determined at various time points. Two weeks later, the levels of serum IL-6 and TNF-α, the mRNA levels of gastrocnemius JAK2 and STAT3, and the expression levels of p-JAK2, p-STAT3, Fbx32, and MuRF1 were examined with ELISA assay, qRT-PCR assay, and Western blotting, respectively. Further studies were performed to assess the levels of Fbx32 and MuRF1 expression and morphological changes.

**Results:**

Pantoprazole can alleviate cancer cachexia-induced body weight reduction and inhibit skeletal muscle wasting in a dose-dependent manner. Our results indicated that pantoprazole treatment can decrease the levels of serum IL-6 and TNF-α (56.3% and 67.6%, respectively), and inhibit the activation of the JAK2/STAT3 signaling pathway. Moreover, the expression levels of MuRF1 and Fbx32 were also suppressed after pantoprazole treatment.

**Conclusion:**

Our findings suggested that pantoprazole can alleviate cancer cachexia skeletal muscle wasting by inhibiting the inflammatory response and blocking the JAK2/STAT3 or ubiquitin proteasome pathway.

## INTRODUCTION

Cancer cachexia is an extremely harmful manifestation of the syndrome and is often associated with body weight loss in patients with malignant tumors. Cancer cachexia is characterized by progressive muscle wasting, usually accompanied by adipose tissue reduction, poor appetite and mental symptoms [1–3]. Muscle wasting is the most clinically relevant feature of cancer cachexia, which results in a life-threatening syndrome associated with poor prognosis and impaired quality of life; notably, blocking muscle wasting can prolong life even in the absence of effects on tumor growth [4], indicating that targeting cancer cachexia could improve outcomes and prolong tumor-free survival. However, the mechanisms underlying cancer cachexia-induced muscle wasting remain poorly understood.

Notably, several studies indicated that inflammatory factors were involved in skeletal muscle loss [5, 6]. The TNF-α serum level may have clinical significance in the development of cancer cachexia [7]. Bonetto et al. also found that IL-6 plays important roles in the skeletal muscle wasting induced by cancer cachexia [8]. Additionally, TNF-α, IL-6, and other inflammatory factors lead to anorexia, the activation of skeletal muscle protein degradation, and the inhibition of protein synthesis; the following systemic inflammatory response aggravates cachexia [9, 10]. Moreover, the JAK/STAT signaling pathway, which is involved in cell proliferation, differentiation, and apoptosis, was also found to play important roles in tumorigenesis and tumor development [11]. A previous study demonstrated that JAK2/STAT3 was also associated with the development of tumor cachexia by inducing a systemic inflammatory response [12]. Another report showed that JAK2/STAT3 can promote skeletal muscle atrophy and degradation by activating the ubiquitin proteasome pathway [13].

Pantoprazole, a proton pump inhibitor, is used to decrease the amount of acid produced in the stomach and treat erosive esophagitis (damage to the esophagus from stomach acid), as well as other conditions involving excess stomach acid such as Zollinger-Ellison syndrome. Recent studies indicated that pantoprazole also has anti-inflammatory, antioxidant, anti-cancer, and chemotherapy sensitization effects [14, 15], which are not associated with its acid suppression, suggesting that it may regulate other unknown signaling pathways. Herein, our purpose of this study was to determine whether pantoprazole can relieve cachexia skeletal muscle wasting and the related mechanisms.

## RESULTS

### The phenotypes of the cachexia group with or without pantoprazole treatment

**Figure 1:**
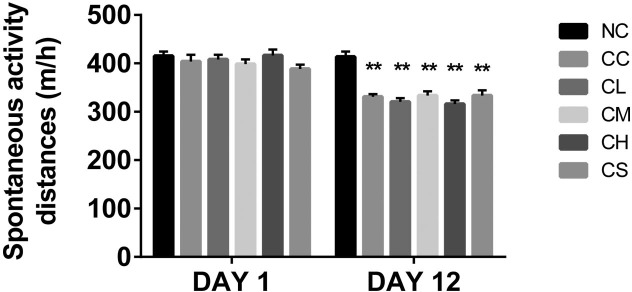
The average distance of the spontaneous activity of mice in different groups at different time points **p<0.01 compared with the combination group.

There was no significant difference in the initial body weight (F=0.8748, P=0.5062) in each group. After 5 days of tumor cell implantation, visible tumors were found in mice implanted with C26 cells. However, the differences of body weight in each group were undetectable. At 12 days post-implantation, the body weight of the mice implanted with the C26 cells were significant decreased compared with that of the mice in the negative control group (p<0.05). Spontaneous activity was also decreased in the C26 cell-implanted mice. Furthermore, other cachexia phenotypes, including matted hair, luster loss, and mental weakness, were observed in these groups [16]. The body weight of the mice in the CC group was significantly lower than that of the NC group (p<0.05). The body weight of the other four groups of pantoprazole-treated mice was dose-dependent (CS<CL<CM<CH) (Figure 1), suggesting that pantoprazole-treatment may alleviate skeletal muscle wasting in cancer cachexia. Our results indicated that the tumor weight had been reduced in the animals in the CH group than those in the CS group (p<0.05; Figure 1), which is consistent with the previous report [15].

### Histological analysis of skeletal muscle wasting in different groups

The weight of the gastrocnemius muscle and the cross-sectional area of muscle fiber were the key parameters for evaluating skeletal muscle wasting. We examined the weight of the right gastrocnemius muscle and measured the muscle fiber cross-sectional areas in each group. The weight of the gastrocnemius muscle as well as the cross-sectional area of muscle fiber were substantially decreased in the CC group compared with those in the NC group (p<0.05). In the pantoprazole-treated groups, including the CL, CM, and CH group, the weight of gastrocnemius muscle and the cross-sectional area of muscle fiber were significantly increased in a dose-dependent manner. Compared with the CS group, the sectional areas of these three groups (CL, CM, and CH groups) were increased by 13.9%, 39%, and 64.9%, respectively (Table 2). This suggested that pantoprazole may be helpful in treating cancer cachexia-induced skeletal muscle wasting.

**Table 1:** The primers for qRT-PCR assay

Gene	Primer sequences
JAK2	5′-CAGCAGCAGAACCTACAGATAC-3′5′-GACAGAGTTATAGACAGCCAGTG-3′
STAT3	5′-TGGCACCTTGGATTGAGAGT-3′5′-TGCTGATAGAGGACATTGGACT-3′

**Table 2:** The body and tumor weight, the weight of the gastrocnemius muscle and the cross-sectional area of muscle fiber in the cachexia group with or without pantoprazole treatment (n=8)

	Groups					
	NC	CC	CL	CM	CH	CS
Original body weight (g)	19.68±0.23	19.43±0.23	19.41±0.32	19.56±0.34	19.44±0.27	19.49±0.32
5-day body weight after inoculation (g)	21.39±0.45	21.18±0.76	21.40±0.51	21.19±0.62	21.41±0.36	21.23±0.54
12-day body weight after inoculation (g)	25.43±0.93	23.81±1.10^*^	24.15±0.72*	23.86±0.90^*^	24.14±0.80*	24.16±0.50*
Tumor weight (g)	—	2.58±0.84	2.21±0.60	2.03±0.46	1.56±0.42*	2.64±1.07
The body weight after tumor removal (g)	31.93±1.37	18.69±1.04^*^	20.69±0.76^*^	23.28±0.89^*^	25.04±1.45**	18.93±1.53
The weight of the right gastrocnemius muscle (mg)	134.84±12.19	66.12±3.79**	73.41±7.30^*^	86.73±3.63^*^	94.81±5.08**	65.80±3.41
The cross-sectional areas of muscle fiber (um2)	2,893.41±175.80	1,283.83±284.25^*^	1,470.83±211.84^*^	1,794.92±136.03^*^	2129.04±198.38^*^	1,291.25±208.03

^*^ *p*<0.05 and ^*^p<0.01 compared with other groups. Each experiment was replicated three times.

### Pantoprazole can regulate the levels of serum inflammatory factors

To elucidate the mechanism by which pantoprazole affects muscle wasting in cancer cachexia, we examined the levels of serum inflammatory factors reported to be involved in skeletal muscle loss during cancer cachexia [5, 6]. The ELISA assay indicated that the levels of serum IL-6 and TNF-α were significantly up-regulated in the CC group compared with the NC group. Additionally, the levels of serum IL-6 and TNF-α were significantly lower in all of the pantoprazole-treated groups (CL, CM, and CH groups) than the CS groups in a dose-dependent manner. The levels of serum IL-6 were reduced by 34.1%, 50.6%, and 67.6%, whereas the levels of serum TNF-α were reduced by 23.2%, 41.2%, and 56.3% in the CL, CM, and CH groups, respectively (Table 3).

**Table 3:** The levels of serum IL-6 and TNF-α in the cachexia group with or without pantoprazole treatment (n=8)

	Groups					
	NC	CC	CL	CM	CH	CS
IL-6 (ng/L)	23.64±1.20	152.18±15.01^*^	100.55±12.48^*^	75.44±11.35^*^	49.50±5.52^*^	152.65±14.10
TNF-α (ng/L)	35.52±3.18	135.40±13.17^*^	96.50±7.17^*^	73.80±4.26^*^	54.83±5.95^*^	125.59±13.94

^*^ *p*<0.05 and ^*^p<0.01 compared with other groups. Each experiment was replicated three times.

### JAK/STAT3 signaling pathway was associated with pantoprazole-alleviated muscle wasting

**Figure 2:**
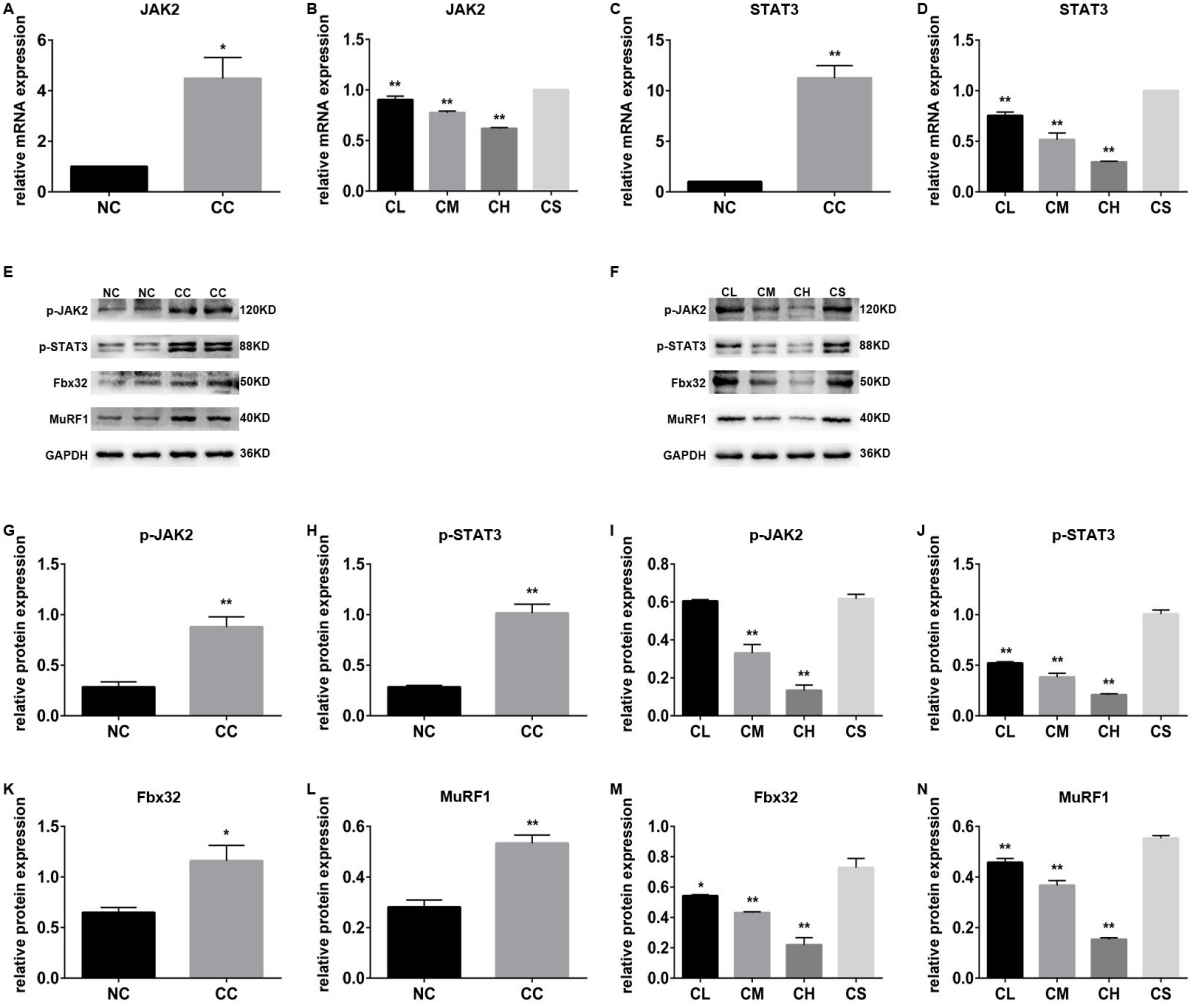
The different expression levels of JAK/STAT3-related proteins in different groups **(A-D)** The mRNA levels of JAK2 and STAT3 in the cachexia group with or without pantoprazole treatment; **(E-N)** the expression levels of p-JAK2, p-STAT3, Fbx32, and MuRF1 proteins in the cachexia group with or without pantoprazole treatment.

A previous study indicated that JAK2/STAT3 was involved in the muscle wasting induced by cancer cachexia by regulating the inflammatory response [12]. We therefore expected that the JAK/STAT3 signaling pathway may also be related to pantoprazole-associated muscle wasting alleviation. As we expected, a further study indicated that the mRNA levels of JAK2 and STAT3 in the CC group were substantially higher than those in the NC group (p<0.05). Moreover, pantoprazole treatment significantly inhibited the mRNA levels of JAK2 and STAT3 in three pantoprazole-treated groups in a dose-dependent manner in the CL, CM, and CH groups, indicating that a higher concentration of pantoprazole has a stronger inhibitory effect on the muscle wasting induced by cancer cachexia (Figure 2A-2D).

Notably, the expression levels of p-JAK2 and p-STAT3 were substantially higher in the CC group than those in the NC group, whereas the expression levels of p-JAK2 and p-STAT3 were remarkably lower in the three pantoprazole-treated groups than the CS group. The expression levels of p-JAK2 and p-STAT3 in the CH group were substantially lower than those in the other groups (p<0.01), which is consistent with its inhibitory effect on the transcriptional levels of JAK2 and STAT3 (Figure 2E-2N).

### Pantoprazole inhibits the ubiquitin proteasome pathway

**Figure 3:**
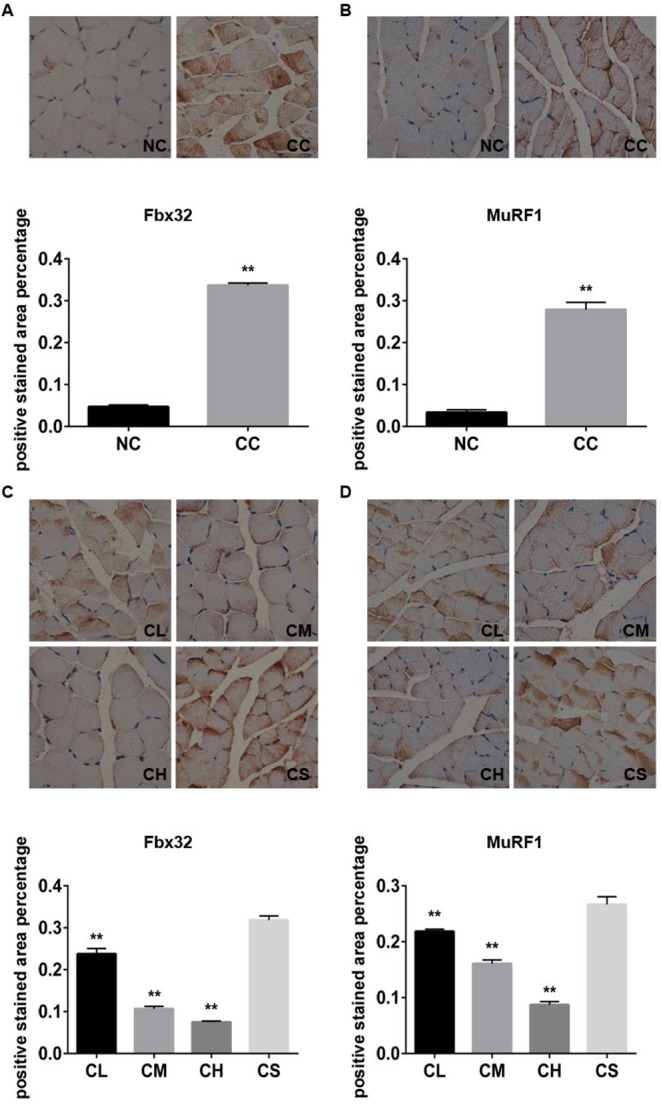
The expression levels of Fbx32 and MuRF1 proteins in different groups The representative images and the percentage of expression levels of Fbx32 **(A)** and MuRF1 **(B)** with immunohistochemistry staining in the NC and CC groups; the representative images and the percentage of expression levels of Fbx32 **(C)** and MuRF1 **(D)** with immunohistochemistry staining in CL, CM, CH, and CS groups.

The ubiquitin proteasome pathway is also involved in JAK/STAT3-related skeletal muscle atrophy and degradation [13]; thus, we next confirmed whether the ubiquitin proteasome pathway plays an important role in this process. The expression levels of MuRF1 and Fbx32, the most two important genes involved in the ubiquitin proteasome pathway, were determined using western blot analysis. Our results indicated that the expression levels of MuRF1 and Fbx32 in the gastrocnemius muscle tissue of CC group were substantially higher than those of the NC group. Similar results were also found by immunohistochemistry assay (p<0.01). Furthermore, western blot analysis and immunohistochemistry demonstrated that pantoprazole treatment can down-regulate the expression of these two proteins in the CL, CM, and CH groups in a dose-dependent manner (Figure 3).

## DISCUSSION

Cachexia is a devastating syndrome associated with many diseases, including cancer, diabetes, and chronic heart failure [17]. Cancer cachexia usually results in lower tolerance for the treatment and subsequently affects the survival rate [1]. Previous reports indicated that over 30% of patients with advanced cancer had died from cancer cachexia [18]. The conventional nutritional supply was not sufficient for treating this syndrome [19]. Cancer cachexia is characterized by progressive muscle wasting [1]. However, the mechanisms responsible for muscle wasting induced by cancer cachexia are not completely known, and a suitable clinical therapeutic strategy for this syndrome remains unavailable.

Pantoprazole is a proton pump inhibitor that is commonly used in the clinical treatment of acid-related diseases. Recent studies demonstrated that the proton pump inhibitors lansoprazole and omeprazole have anti-inflammatory, anti-tumor, and chemotherapy functions [14, 20–22], which are critically associated with the muscle wasting induced by cancer cachexia. Therefore, the present study explored the anti-inflammatory effects of pantoprazole (which belongs to a subgroup of benzimidazole proton pump inhibitors that includes omeprazole and lansoprazole) and clarified the mechanism of action in the treatment of cancer cachexia-induced muscle wasting.

IL-6, TNF-α, and other inflammatory factors were found to play important roles in cancer cachexia skeletal muscle atrophy, which leads to anorexia, the activation of skeletal muscle protein degradation, and protein synthesis inhibition and can aggravate the process of cachexia through the systemic inflammatory response [9, 10]. Our results also indicated that the levels of serum IL-6 and TNF-α were decreased after the treatment of pantoprazole in a dose-dependent manner in cachectic mice, suggesting that the effects of pantoprazole on the alleviation of muscle wasting induced by cancer cachexia may be associated with its inhibitory effects on the regulation of the serum levels of inflammatory factors.

The JAK/STAT pathway is primarily composed of the JAKs and STATs protein family. JAK is usually stimulated by the extracellular cytokine signal and regulates the transcription of certain genes involved in cell stress, proliferation, differentiation, and apoptosis. Interestingly, a recent study indicated that the activation of the JAK2/STAT3 signaling pathway plays an important role in cancer cachexia [23], and the use of AG490, a JAK2 specific inhibitor, can improve the prognosis of cancer cachexia [24]. Furthermore, previous reports indicated that pantoprazole can inhibit astrocytes and gastric cancer cells as well as the phosphorylation level of STAT3 [25, 26]. As we expected, our results also showed increased transcriptional levels and phosphorylation levels of JAK2 and STAT3 in the gastrocnemius muscle tissue of cachectic mice. We also found that pantoprazole inhibits the activation of the JAK2/STAT3 pathway and that a higher concentration of pantoprazole treatment displayed better efficacy in treating the muscle wasting induced by cancer cachexia by regulating the JAK2/STAT3 pathway.

We also found that the regulation of MuRF1 and Fbx32, important factors in the ubiquitin proteasome pathway [27, 28], were associated with muscle atrophy progression [29]. Therefore, the possible mechanisms of pantoprazole treatment for cancer cachexia-induced muscle wasting may include the following steps. (1) pantoprazole can significantly inhibit the levels of serum IL-6 and TNF-α in cachectic mice, which may induce a local and systemic inflammatory response; (2) the treatment of pantoprazole also suppresses the systemic inflammatory response-induced activation of the JAK2/STAT3 signaling pathway [12]; (3) the inactivation of the JAK2/STAT3 signaling pathway subsequently inhibits the expression of MuRF1 and Fbx32 protein, which can significantly alleviate the progression of tumor cachexia-related muscle atrophy and protein degradation [30].

In summary, pantoprazole could effectively alleviate cancer cachexia-induced skeletal muscle wasting, and a higher concentration of pantoprazole treatment had a stronger effect in treating muscle wasting. We found that the effects of pantoprazole on cancer cachexia-induced muscle wasting may be associated with the inhibition of inflammatory cytokines and/or the inactivation of the JAK2/STAT3 and ubiquitin proteasome pathways. Our results also demonstrated that pantoprazole treatment with a high concentration has inhibitory effects in a cancer cachectic mice model. However, whether pantoprazole treatment can prevent the skeletal muscle wasting of cachexia *via* other signaling pathway remains to be clarified. Therefore, our findings may provide a new strategy for the clinical treatment of cachexia-induced muscle wasting.

## MATERIALS AND METHODS

### Cells line and culture

Mice colon adenocarcinoma cell line C26 was obtained provided by the Experimental Research Center of the First Affiliated Hospital of Chongqing Medical University (Chongqing, China). The cell line was maintained in DMEM/F12 medium (Gibco, Waltham, MA, USA) with 10% fetal bovine serum (FBS; Gibco) and 1:100 penicillin/streptomycin (Invitrogen, Carlsbad, CA, USA) in a humidified incubator with 5% CO_2_ at 37°C.

### Animal treatment

Male BALB/C (17-19 g; 6-8 weeks) mice were obtained from the Experimental Animal Center of Chongqing Medical University. The animals were housed four animals per cage in standard polycarbonate cages with free access to food and water with a 12/12 hrs light/dark cycle and a temperature-regulated environment (23 ± 1°C) for 7 days.

The mice were randomly divided into 5 groups, including a cachexia group (CC group), lower dose pantoprazole-treated (Nuoweinuo Company, Liaoning, China) group (37.5 mg/kg; CL group), medium-dose pantoprazole-treated group (75 mg/kg; CM) [31], higher dose pantoprazole-treated group (150 mg/kg; CH), and saline group (CS). The NC and CC groups of mice were housed with free access to food and no drug treatment, whereas the mice in the CL, CM, CH, and CS groups were treated with different drugs *via* intragastric gavage (100 μl/10 g). The pantoprazole treatment was performed at 13 days after the inoculation of tumor cells in cachectic mice. The mouse hair, mental state, body weight, tumor growth, and spontaneous activity were determined daily. Spontaneous physical activity was monitored using an infrared monitoring system (WV-CF314LCH, Panasonic, Japan) [32].

### Tumor inoculation assay in mice

All animal experimental procedures were reviewed and approved by the Institutional Animal Care and Use Committee of Chongqing Medical University. 1×10^6^ C26 cell suspensions in 100 μl PBS were injected subcutaneously into the skin under the front legs of the mouse as the experiment group (n=40) [16]. A subcutaneous injection of PBS buffer in the same parts of 8 mice served as a negative control (NC).

### Sample preparation

After 2 weeks of drug treatment, each mouse received an intraperitoneal injection of pentobarbital sodium for anesthesia, and the blood sample was collected after eye removal. The samples were centrifuged at 4,000 rpm for 10 min and the supernatant was removed to a fresh tube and frozen at −20°C. The mice were then euthanized by cervical dislocation. The gastrocnemius muscle of the right lower extremity was then assessed using Western blot analysis and immunohistochemistry.

### Histological analysis

The gastrocnemius muscle of the right lower extremity was removed, fixed in 10% buffered formalin and paraffin-embedded. The 5-μm-sections were stained with hematoxylin and eosin (H&E). The H&E stained slides were observed and evaluated under light microscopy for histological examination. The photographs were analyzed with ImageJ software (NIH, Bethesda, USA). Each sample was randomly selected to calculate the average cross cutting area of 200 muscle fibers [33].

### ELISA assay

The serum levels of IL-6 and TNF-α were determined with an ELISA assay kit (Cloud-Clone Corp., Wuhan, Hubei Province, China) with standard protocol. In brief, the serum supernatant was harvested after centrifugation and then diluted with dilution solution. A total of 100 μl of the diluted serum supernatant was used to coat each well of 96-well plates, and the plates were incubated at 37°C for 90 min. After washing with PBS three times, the plates were incubated the corresponding primary antibody for 60 min at room temperature. After washing with PBS three times again, the plates were then incubated with a SABC solution at 37°C for 30 min. TMB color solution was then added for 15 min at room temperature. The Optical density (OD) was then read with a spectrophotometer at 450 nm. Following drawing the standard curve of IL-6 and TNF-α concentrations, the levels of serum IL-6 and TNF-α were quantified and the data expressed as the means ± SD. The experiments were repeated at least three times.

### qRT-PCR assay

Total RNA from the frozen gastrocnemius muscle tissue was extracted using Trizol reagent (TaKaRa, Dalian, Liaoning, China) according to the manufacturer's instructions. qRT-PCR was performed using the SYBR Green quantitative reverse transcription PCR system. Each qRT-PCR reaction (10 μl) included 1 μl of diluted cDNA, 0.6 μl of primers (Table 1), 5 μl of SYBR fast qPCR master mix, and double distilled water. The PCR comprised an initial denaturation at 95°C for 1 min, then 40 cycles at 95 C for 5 sec, 58°C for 15 sec, and 72°C for 15 sec. The relative quantification of gene expression was performed using the comparative CT method (2^−ΔΔCt^ method). Each sample was replicated at least three times.

### Western blot

Total protein was extracted in a lysis buffer from Pierce (Rockford, IL, USA) on ice for 30 min. After centrifugation, the protein concentration in the supernatants was determined by the BCA protein assay kit (Beyotime, Shanghai, China), and 20 μg of each sample was separated in 10% SDS-PAGE and then transferred onto PVDF membranes (Millipore, Billerica, MA, USA). The signals were detected with anti-p-JAK2 (1:2,000; Abcam, Cambridge, UK), p-STAT3 (1:2,000; Abcam), Fbx32 (1:2,000; Proteintech Group, Inc., Rosemont, IL, USA), MuRF1 (Proteintech Group, Inc.), and GAPDH (1:3,000; Santa Cruz Technology, Santa Cruz, CA, USA), respectively.

### Immunohistochemistry (IHC) staining

The immunohistochemistry experiment in this study had modified protocols. The samples were incubated with the indicated primary antibody (1:100) at 4°C overnight and then rinsed with PBS for 5 min three times and incubated with biotinylated secondary antibody (1:100) at room temperature for 30 min. After PBS washing, DAB was then added for color development. The reaction was controlled under a microscope. When the color was fully developed, the sample was rinsed with PBS thoroughly to quench the reaction. The sample was then patched, dehydrated, cleared, and mounted with neutral gum. Image analysis was performed using Pro Plus Image software (Media Cybernetics, Inc., Bethesda, MD, USA) to determine the percentage of Fbx32- and MuRF1-positive staining area.

### Statistical analysis

The statistical analysis was performed using the SPSS 17.0 software package (SPSS, Chicago, IL, USA). The data are expressed as the means ± standard deviation (x¯±s). Student's t test was used for raw data analysis. One-way ANOVA was performed when more than two animal groups were compared. Comparisons of multiple groups were performed using Tukey's multiple comparisons test. Multiple comparisons between multiple groups and a single group were analyzed using Dunnett's multiple comparisons test. *p*<0.05 was considered to be statistically significant.

Chaoyi Wang contributed significantly to analysis and manuscript preparation;

Dunwei Guo performed the data analyses and wrote the manuscript;

Qiang Wang and Zhongpeng Qiao helped perform the analysis with constructive discussions.
